# Dehydrated at Different Conditions and Powdered Leek as a Concentrate of Biologically Active Substances: Antioxidant Activity and Phenolic Compound Profile

**DOI:** 10.3390/ma14206127

**Published:** 2021-10-15

**Authors:** Beata Biernacka, Dariusz Dziki, Joanna Kozłowska, Iwona Kowalska, Agata Soluch

**Affiliations:** 1 Department of Thermal Technology and Food Process Engineering, University of Life Sciences in Lublin, 31 Głęboka St., 20-612 Lublin, Poland; beata.biernacka@up.lublin.pl; 2 Department of Chemistry, Faculty of Biotechnology and Food Science, Wrocław University of Environmental and Life Sciences, 25 Norwida St., 50-375 Wrocław, Poland; joanna.kozlowska@upwr.edu.pl; 3 Department of Biochemistry and Crop Quality, Institute of Soil Science and Plant Cultivation-State Research Institute, 8 Czartoryskich St., 24-100 Puławy, Poland; ikowalska@iung.pulawy.pl (I.K.); asoluch@iung.pulawy.pl (A.S.)

**Keywords:** leek, freeze-drying, vacuum drying, air-drying, antioxidant activity, phenolic compound profile, color, powder

## Abstract

This study aimed to analyze the antioxidant activity, phenolic acid profile, color changes, and chemical composition of dried and powdered leek (*Allium porrum*). Leek was divided into white shaft (WH) and green shaft (GR) and subjected to drying by different methods—convection drying, vacuum drying, and freeze-drying (FD)—at a temperature of 60 °C. A sample freeze-dried at a temperature of 20 °C was used as control. Analyses of the dried leek samples revealed that GR contained a higher amount of ash, protein, fat, fiber, phenolic acids, and flavonoids, and exhibited higher antioxidant capacity compared to WH. The dominant phenolic acid in WH was *p*-cumaric acid followed by synapic and protocatechuic acids. GR had a several-fold higher content of phenolic acids than WH, with ferulic acid being dominant (about 85% of the total phenolic content). It was also observed that a higher drying temperature resulted in the degradation of phenolic compounds and reduced the antioxidant properties of leek shafts. Most importantly, FD under a temperature of 60 °C caused a similar degree of degradation of biologically active compounds as air drying. An increase in drying temperature was associated with a slight decrease in the lightness of GR, whereas in the case of WH no significant change in this parameter was observed.

## 1. Introduction

Leek (*Allium porrum*) is a biennial plant belonging to the garlic family (amaryllis). It originated from Asia Minor and was cultivated for consumption in ancient Egypt, Greece, and Rome. Its edible shaft is onion and leaves. Initially, leek was used as an appetizer or as a thickener for soups, but over time it came to be served in a cooked form with olive oil. People also developed an interest in the medicinal properties of leek and used it for insect bites, tuberculosis, and even hemorrhages [1]. The inflorescence shoot of this plant can grow up to a height of 200 cm [2]. The unique bicolor of the stem is attributed to the presence of essential oils in different amounts. The white shaft (WH) of leek has a milder flavor, while the green shaft (GH) has a spicy taste [3].

Similar to other broth vegetables, leek is low in calories (100 g contains only 25 calories) [1]. Its spicy taste is reminiscent of onion or garlic, but the vegetable is easily softened by cooking [4]. Leek is rich in minerals such as zinc, iron, calcium, phosphorus, copper, potassium, sodium, manganese, magnesium, and vitamins such as A (carotene), B (B1, B2, B3, and B6), C, E, and K as well as folic acid [5]. In addition, it contains many antioxidants, such as lutein and zeaxanthin, nicotinic acid, vegetable protein, as well as fats, carbohydrates, fiber, sugars, sulfide oil, and sulfur compounds exhibiting bactericidal properties [6]. Leek also has several biologically active compounds such as polyphenols, glucosinolates, and pectic polysaccharides, and S-alkenyl-l-cysteine sulfoxides, a metabolite [7]. Thus, its inclusion in the diet can aid in protecting blood vessels and blood cells from oxidative damage [4]. Although this plant contains a lesser amount of polyphenols than, for example, garlic or onion, it is easily digestible and promotes proper functioning of the body [8]. Moreover, leek has been shown to decrease the risk of different cancers [9,10]. Furthermore, the extracts of *Allium ampeloprasum* show antiallergic and anti-inflammatory effects [11]. Leek has also been found to exhibit antimicrobial properties [7].

The durability of plant raw materials determines the need for their processing and management. Drying is one of the main methods applied for food preservation. However, it can cause a number of undesirable changes [12]. In industries, drying is a basic process used to obtain products and substrates, extend the lifetime of a given material, and improve its strength. The process removes or reduces the water content of a solid body by transferring it to a drying agent [13,14]. Application of appropriate drying parameters, such as heat delivery method, process temperature, and drying air flow speed, can result in products with desirable structural features, color, aroma, and nutrients or antioxidants [15]. Due to their health-promoting properties, plant materials are dried in such a way that unfavorable physical and chemical changes related to dehydration are limited [16]. Dried and powdered leek can be used as a functional food additive [17,18,19,20]. However, the characteristics of powdered leek obtained under different drying conditions has not been studied so far. Therefore, this study aimed to assess the phenolic acid profile and antioxidant properties of WH and GR of leek dehydrated in different conditions.

## 2. Materials and Methods

### 2.1. Materials

All chemicals used in the study were of analytical grade. Methanol and acetonitrile of high-performance liquid chromatography (HPLC) grade and formic acid of liquid chromatography–mass spectrometry (LC–MS) grade were purchased from Merck (Darmstadt, Germany). Ultrapure water was obtained in-house using a purification system (Milli-Q-Simplicity-185, Millipore Corp, Molsheim, France). Reference standard kaempferol was purchased from Fluka AG (Buchs, Switzerland). Other chemicals were purchased from Sigma Aldrich (St. Louis, MO, USA). Leek (*A. porrum*, cv. Bartek) was collected from Lublin (Poland) region in autumn 2019.

### 2.2. Sample Preparation

The collected plant material was thoroughly cleaned, stripped of the root shaft, and divided into WH and GR portions. Then, they were cut into discs with a thickness of about 0.5 cm. The prepared raw material was then subjected to drying.

Drying was carried out by three different methods: convection drying, vacuum drying (VD), and freeze-drying (FD). Convection drying was carried out in a laboratory convection dryer (Promis Tech, Wroclaw, Poland) at an air temperature of 60 °C and a constant air flow velocity of 0.5 m/s. FD and VD processes were carried out in a laboratory vacuum dryer (ALPHA 1–4, Martin Christ Gefriertrocknungsanlagen GmbH, Osterode am Harz, Germany). For FD, the heating plates in the dryer were maintained at temperatures of 20 °C and 60 °C at a constant pressure value of 63 Pa [21]. A sample freeze-dried at a temperature of 20 °C was used as control (CS). Before FD, leek was frozen at −30 °C in a freezer (Liebherr GTL-4905, Bulle, Switzerland) for 24 h.

For VD, the heating plates of the dryer were maintained at a temperature of 60 °C at a constant pressure value of 2000 Pa (the material was not previously frozen).

The initial mass of each dried sample was 200 g. All dryers were equipped with a mass recording system [21,22]. Drying was carried until the final moisture content in the sample was 10–11%. The time taken for drying was determined. Three independent dryings were performed for each sample. The dried leek samples were powdered using a knife grinder (Retsh, Grindomix GM 200, Düsseldorf, Germany), and particles below 350 µm were selected for further analyses.

### 2.3. Determination of the Basic Chemical Composition

The WH and GR of fresh and dried leek samples were chemically analyzed to determine the content of dry matter, crude protein, crude ash, and crude fat using Method 44-15A, Method 08-01, Method 46-06, and Method 30-10, respectively. The analysis was performed in triplicate and in accordance with the AOAC standards [23].

### 2.4. Color Coordinate Measurements

The color coordinates of dried and powdered leek samples were determined in the CIEL*a*b* system, using a colorimeter (CR-400C Chroma Metre 115, Minolta, Colour Lab, Osaka, Japan). The CIEL*a*b* system consists of coordinates L*, a*, and b*. Of these, L* denotes lightness and ranges from 0 (perfect black body) to 100 (perfect white body). The a* coordinate specifies the change of green color (−a*) to red color (a*), while b* determines the change of color from blue (−b*) to yellow (b*) [24].

### 2.5. Quantitative and Qualitative Analyses of Phenolic Compounds

#### 2.5.1. Flavonoids Determination

For the determination of flavonoids, the plant material was first defatted with chloroform in a Soxhlet apparatus and extracted using an automatic extractor (Dionex ASE 200 Accelerated Solvent Extraction System, Sunnyvale, CA, USA) under the following conditions in three cycles: extraction solvent—80% methanol, solvent pressure—1500 psi, and temperature of cell extraction—40 °C. After extraction, the samples were evaporated to dryness in a rotary evaporator (Heidolph Hei-Vap Advantage, Schwabach, Germany) under reduced pressure at 40 °C. Then, the extracts were dissolved in 1 mL of 75% methanol and transferred quantitatively to 2 mL Eppendorf tubes. The final concentration of samples was 200 mg dm/mL. The extracts were maintained at −20 °C. Prior to the LC–MS/MS analysis, they were sonicated for 5 min at 25 °C (SONOREX DIGITEC DT 510 H, Bandelin, Germany) and centrifuged for 10 min at 10,000 rpm (Polygen Sigma 3–16 KL laboratory centrifuge, Sigma, Darmstadt, Germany). Finally, 150 μL of supernatant was collected from each sample and subjected to analysis.

Ten flavonoids were identified and quantified by an Acquity UPLC system, equipped with a photodiode array (PDA) and a triple quadrupole mass detector (TQD, Waters, Milford, MA, USA). The compounds were separated using an Acquity UPLC BEH C18 column (100 mm × 2.1 mm, 1.8 µm particle size; Waters, Manchester, UK) with a gradient mobile phase consisting of water (solvent A) with 0.1% formic acid and acetonitrile (solvent B) with 0.1% formic acid as follows: 5–30.4% in 12 min, 30.4–99% from 12 to 12.1 min, 99–5% from 12.1 to 15.1 min, and finally 5% in 17 min. The flow rate was set to 0.5 mL/min, column temperature was maintained at 50 °C, and injection volume was 2.5 μL. Compounds were interpreted based on the mass spectral data. Electrospray ionization (ESI) was performed in negative ion mode. The ion source parameters were as follows: capillary voltage—2.8 kV and cone voltage—40 V. The source and desolvation temperatures were maintained at 150 °C and 450 °C, respectively. The flow of collision gas used as cone was set to 100 L/h and the flow of desolvation gas to 800 L/h. PDA was operated at 191–480 nm, with a resolution of 3.6 nm. Data processing was performed using MassLynx V4.1 software (Waters, Version 4.1, Manufacturer, Milford, MA, USA).

#### 2.5.2. Phenolics Acids Determination

For the determination of phenolic acids, leek samples were prepared according to the modified method of Żuchowski et al. [25]. Briefly, samples (200 mg) were hydrolyzed with 4 N NaOH and 2% ascorbic acid solution for 4 h at room temperature. Then, they were cooled in ice and acidified with ice-cold 6 M HCl until the pH value was 2. The resulting mixtures were centrifuged at 8000 rpm (Sigma 2–16) for 20 min. The supernatants were collected and extracted thrice with ethyl acetate. Then, the organic phase was removed, filtered, and evaporated to dryness at 35 °C in a rotary evaporator. The obtained residue was dissolved in 30% methanol and stored in a freezer.

Analysis was performed in an Acquity UPLC system, equipped with a PDA and a TQD (Waters, Milford, MA, USA). Samples were separated on a Waters Acquity UPLC HSS C18 column (100 mm × 2.1 mm, 1.8 µm), using a mobile phase consisting of solvent A (acidified water, 0.1% formic acid) and solvent B (acidified acetonitrile, 0.1% formic acid). The solvent gradient was programmed as follows: 8–20% B in 8 min, 20–95% B in 2.9 min, and 95–8% B in 2 min. The flow rate was set to 0.5 mL/min, column temperature was maintained at 30 °C, and injection volume was 2.5 μL. The compounds were interpreted based on the mass spectral data. ESI was performed in negative ion mode. Data processing was performed using MassLynx V4.1 software (Waters). The results were expressed per g dm.

### 2.6. Antioxidant Properties

For the preparation of extracts, the ground dried sample was extracted with 50% methanol (shaken thrice for 30 min). The homogenate was centrifuged at 4000 rpm for 10 min at a temperature of 4 °C [26]. Extraction was performed twice. The obtained supernatants were combined and used for further analyses.

Antioxidant activity (AA) of the extracts was examined by analyzing:

- The ability to scavenge ABTS (2,2′-azinobis (3-ethylbenzothiazoline-6-sulfonate) free radicals using the method of Re et al. [27];
- The ability to neutralize DPPH (2,2-diphenyl-1-picrylhydrazyl) free radicals as described by Brand-Williams et al. [28];
- The chelating power (CHEL) by determining chelate metal ions as described by Guo et al. [29];
- The reducing power (RED) as described by [30].

The AA of the extracts was expressed by the EC_50_ index [31], which refers to the half-maximal effective concentration of antioxidants that causes a 50% decrease in activity. 

### 2.7. Statistical Analysis

Three independent dryings were performed for each sample and all tests were performed for each sample (three repetitions). Mean values and standard deviations and means were calculated. One-way and two-way analysis of variance (ANOVA) and linear correlation analysis were also performed, and significant differences between means were determined by Tukey’s test using Statistica software, (version 13.1, TIBCO Software, Palo Alto, CA, USA). All calculations were performed at a significance level of *α* = 0.05.

## 3. Results and Discussion

### 3.1. Drying Time

**Figure 1:**
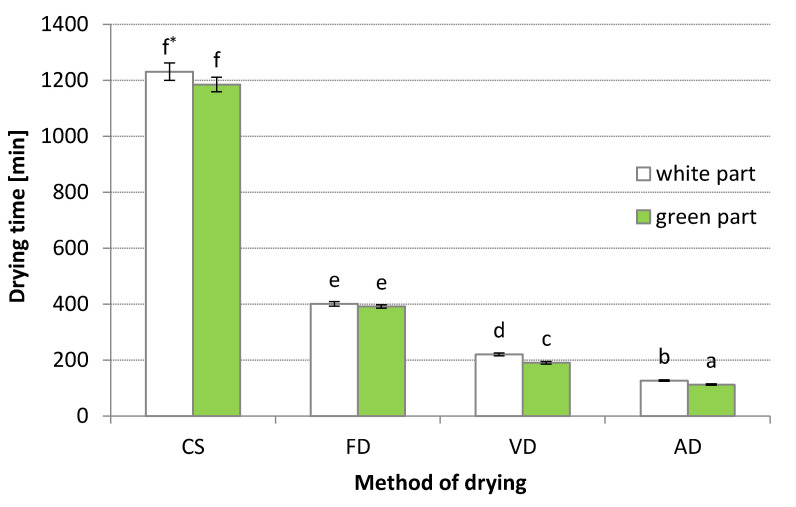
Drying time taken to achieve final moisture content of 10–11% using different methods in green and white shaft of leek: CS—control sample, FD—freeze-drying, VD—vacuum drying, AD—air drying; * The values designated by the different small letters significantly different (α = 0.05).

The estimated drying time (DT) of leek samples is presented in Figure 1. An increase in FD temperature from 20 °C to 60 °C reduced the DT by about three-fold for both WH and GR of leek (on average from 1208 to 396 min). A similar three-fold reduction in DT has been observed for kale when the temperature of heating plates in the freeze-dryer was increased from 20 °C to 60 °C [32]. When the same drying temperature was applied, DT was shorter than that of air-drying (AD): 127 and 112 min for WH and GR of leek, respectively. The longest dehydration period was found for FD. For VD, the dehydration time was about 200 min for both shafts of leek. The DT for both WH and GR of leek was similar. Only in the case of VD and AD, a statistically significant but slightly shorter DT was observed for GR compared to WH. Other authors also observed that FD takes much longer than other drying methods [33].

The results of the variance analysis of the DT are presented in Table 1. The part of the leek (WH or GR) and drying method (DM) had a significant influence on the DT. The interaction between leek sample and DM was insignificant. According to the value obtained by the F-test, the DM had the highest influence on DT. 

### 3.2. Results of the Analysis of Basic Chemical Composition

The content of dry matter in the WH and GR of fresh leek was 12.44% and 11.85%, respectively (Table 2). The GR of fresh leek samples was characterized by higher content of ash, fat, and fiber compared to WH. After drying, the content of dry matter increased up to 88.69% and 88.55% in WH and GR, respectively. The dehydration method was found to have a little or no influence on ash, protein, fat, and fiber content in leek samples. The average mass fractions of these compounds in GR were 5.16%, 12.37%, 2.26%, and 8.28%, respectively. In the case of WH of dried leek, the content of ash, fat, and fiber was lower and estimated at 4.80%, 1.26%, and 5.03% on average, respectively. 

### 3.3. Color Coordinates

During thermal processing, especially depending on the drying conditions applied, the structural characteristics of natural pigments in plant material and the color of products usually change [13,17]. In the WH and GR of CS, the average values of color parameters were as follows: L* = 85.79, a* = −3.44, b* = 22.15 and L* = 76.22, a* = −9.32, b* = 27.07, respectively (Table 3). The results of the analysis of the variance of the color parameters were presented in Table 4. Both the drying method and the part of the leek had a significant impact on the color parameters. Additionally, the interaction between these factors was statistically significant for all color coordinates. However, the strongest influence on color was the part of the dried leek. An increase in drying temperature caused a slight decrease in the lightness of GR. The highest decrease in this parameter was observed during AD (L* = 70.98) and the lowest during VD (L* = 74.17). On the other hand, lightness did not change significantly in WH. The highest greenness and yellowness of GR were observed in freeze-dried leek (a* = −8.96 and b* = 29.05), whereas air-dried leek was characterized by the lowest values of these parameters (a* = −7.14 and b* = 26.8). In the case of powdered WH, a decrease in greenness and yellowness was observed with an increase in drying temperature during VD and AD. An increase in air-drying temperature usually leads to higher color changes in dried products [34] and causes a destruction of most valuable nutrients [35]. Importantly, a higher temperature during FD had no significant influence on the a* and b* parameters of WH samples. In general, compared to raw material, unfavorable changes in the color of vegetable-based products mainly result from the degradation of compounds such as chlorophyll, which causes brownish discoloration of dried material [31,36]. Moreover, the presence of oxygen, especially during AD, leads to the oxidation of unsaturated pigments contained in the material [36], contributing to changes in color determinants. Therefore, FD and VD are highly recommended to obtain good quality vegatable powders rich in bioactive compounds [37,38].

### 3.4. Results of Quantitative and Qualitative Analyses of Phenolic Compounds

**Figure 2:**
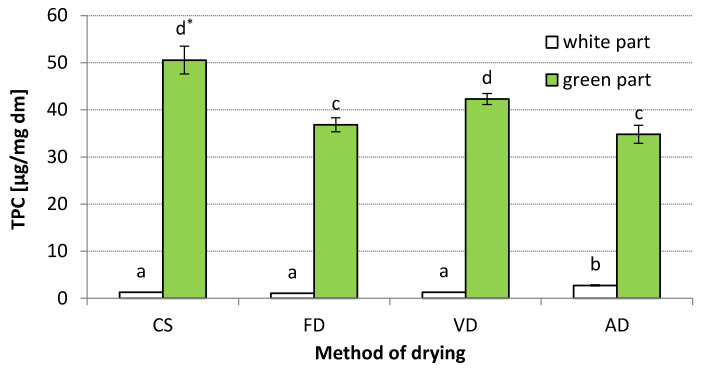
Total phenolic content in dried leek samples: CS—control sample, FD—freeze-drying, VD—vacuum drying, AD—air drying, WH—white shaft, GR—green shaft; * The values designated by the different small letters are significantly different (α = 0.05).

The content of total phenolic compounds in dried leek samples is presented in Figure 2. TPC was found to vary in WH and GR and significantly influenced by drying methods. Additionally, the interection between DM and leek sample was significant (Table 5). In the WH of CS, the average TPC was 1.3 µg/mg dm, while in GR it was several-fold higher (50.6 µg/mg dm). Kovarovič et al. [3] also found that GR is characterized by a higher content of total phenolic compared to WH. Our results are lower compared to Kovarovič et al. [3], who found that the content of total phenolics depending on leek variety ranged from 504 to 1117 µg/mg dm, and from 777 to 1224 µg/mg dm for WH and GR, respectively. However, they determined the total polyphenols content for fresh leek and used a different method (Folin–Ciocalteau assay).

An increase in drying temperature significantly reduced TPC in the GR of leek. Interestingly, at 60 °C, FD and VD caused a similar level of degradation in total phenolic compounds (TPC decreased to 36.84 and 42.30 µg/mg dm). Importantly, FD at higher temperatures led to a similar extent of reduction in TPC as AD (decrease was 27.1%). It is also worth emphasizing that compared to AD, the time of dehydration with FD was four-fold longer at the same temperature (60 °C). Although lyophilisation at a higher temperature significantly reduces the FD time and give appropriate sensory quality, it also often has a more destructive effect on phytochemicals [39,40]. Drying temperature and TD are crucial factors influencing the biodegradation of phenolic compounds. Usually, an increase in drying temperature leads to a higher degree of degradation of phenolic compounds. Previous studies have confirmed that longer DTs and higher temperatures were associated with a loss of phenolic compounds in *A. porrum* vegetables [41]. However, the application of higher temperature for a shorter time may also have a beneficial effect in preserving phenolic compounds in vegetables [42,43].

The phenolic acids profile of dried leek samples is presented in Table 6 and analysis of variance of obtained results is included in Table 7. Taking into account the results of variance analysis, it can be observed that both DM and SA of the leek have significant influence on all phenolic acids content. Moreover, in the case of all of the phenolic acids, significant interactions were found between DM and SA. In the WH of leek, *p*-cumaric acid was the dominant phenolic acid, followed by synapic and protocatechuic acids. *p*-Hydroxybenzoic, ferulic, and protocatechuic acids were also detected in small amounts in dried WH samples. Compared to WH, the content of phenolic acids was several times higher in the GR of leek. Ferulic acid was the dominant phenolic acid in GR (about 85% of TPC), followed by *p*-cumaric (10.2%) and synapic acids (3.6%). *p*-Hydroxybenzoic, protocatechuic, and caffeic acid were also detected in dried GR. Ferulic acid offers many health benefits and exhibits antioxidant, anti-inflammatory, anticancer, and antimicrobial activities [44]. Hence, it has been widely used in food [45], cosmetic [46], and pharmaceutical industries [44]. Some studies have also shown that food rich in this acid might prevent hypertension [47]. In addition, ferulic acid shows promise in remedying vascular disorders [48]. When leek samples were dried at 60 °C, a significant reduction in TPC was noted in GR. The highest reduction in ferulic acid (decrease of about 38%) was observed during dehydration by AD. On the other hand, a lower rate of reduction was observed when FD and VD were applied (25.2% and 16.7%, respectively). Interestingly, FD and VD also caused a significant decrease in the content of *p*-coumaric and synapic acid, while AD had only a mild reducing effect on these acids.

**Figure 3:**
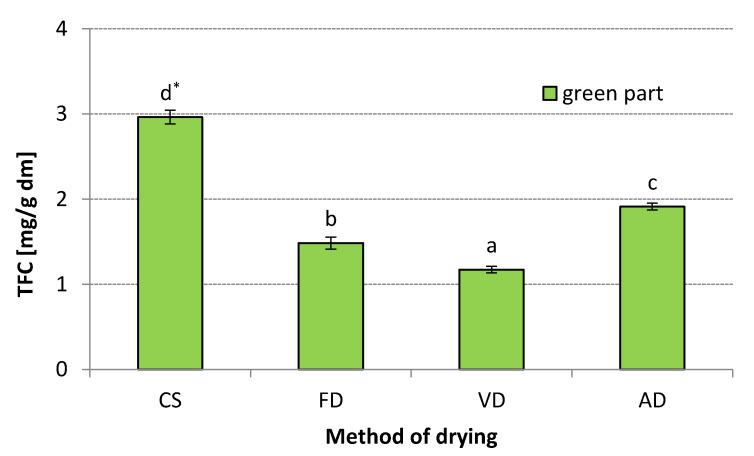
Total flavonoid content (mg/g dm): CS—control sample, FD—freeze-drying, VD—vacuum drying, AD—air drying * The values designated by the different small letters are significantly different (α = 0.05).

Fruits and vegetables, as well as spices, are an essential part of the human diet. They are rich in natural antioxidants, mostly flavonoids, which helps in the maintenance of normal physiological functions [49]. A large part of flavonoids exist as dimers and oligomers and contain C-glycosides. During industrial processing, application of heat causes the hydrolysis of glycosides and results in the release of flavonoid monomers [36,50]. The total flavonoid content (TFC) also varies depending on the part of the leek from which the extracts were prepared (Figure 3). In this study, flavonoids were not detected in the WH of leek, whereas the TFC in the GR ranged from 1.174 to 2.96 mg/g dm, respectively, when VD and CS were applied for drying. Drying at 60 °C caused a significant reduction in TFC in leek samples. The highest decrease was found in vacuum-dried and freeze-dried samples (decrease up to 1.174 and 1.485 mg/g dm, respectively). The lowest reduction in TFC was found when the leek samples were air-dried. The average content of TFC in the GR of leek estimated after AD was 1.91 mg/g dm. Many authors have shown that FD compared to AD is superior in maintaining phytochemicals in plant materials after dehydration [51,52,53]. However, according to this study application of FD at higher temperatures (the same as AD) led to a higher degree of degradation of flavonoids as AD (decrease up to 49.62% and 35.47%, respectively).

*Allium* species, including onion (*Allium cepa* L.), garlic (*Allium sativum* L.), chives (*Allium schoenoprasum* L.), leek (*A. porrum* L.), and wild garlic (*Allium ursinum* L.), are extensively used as flavoring agents in food. They are also an excellent source of secondary metabolites, such as polyphenolic compounds (phenolic acids and their derivatives), flavonoids (flavan, flavanone, flavones, flavonol, dihydroflavonol, flavan-3-ol, flavan-4-ol, and flavan-3,4-diol), and flavonoid polymers (proanthocyanidins or condensed tannins) [4]. In the present study, qualitative and quantitative analyses of leek samples revealed that flavonoid I–X compounds (kaempferol derivatives) were present only in dried GR (Table 8). The presence of kaempferol derivatives in leek has also been confirmed in previous studies [4,49]. These compounds possess many prohealth properties, especially antimicrobial and anticancer activities [54]. In the control leek sample, the dominant kaempferol derivative was acylated kaempferol glycoside, followed by kaempferol derivative and kaempferol glycoside (1.038, 0.748, and 0.303 mg/g dm, respectively). A significant amount of acylated kaempferol glycoside (0.501 mg/g dm) and kaempferol derivative (0.295 mg/g dm) was also detected in some freeze-dried samples, while only acylated kaempferol glycoside was detected in vacuum-dried samples (0.467 mg/g dm). In the air-dried sample, acylated kaempferol glycoside was the dominant flavonoid (about 27.5% of TFC), followed by kaempferol derivative (21.1%). Drying at 60 °C caused a significant reduction in the content of kaempferol derivatives in leek samples, and the highest reduction (four derivatives of the compounds were below the limit of quantification) was observed with dehydration by VD. A lower reduction in kaempferol derivatives was observed with FD and AD at the same temperature.

### 3.5. Antioxidant Properties

Determination of AA of food has been attracting increasing interest. The beneficial influence of fruits, vegetables, and cereals on human health seems to be related to their antioxidant properties [4]. In this study, analysis of the AA of extracts obtained from dried and powdered leek samples showed that the drying methods had a significant influence on the ABTS and DPPH scavenging activity, CHEL, and RED, and significant differences in these properties were found in the parts of leek studied (WH and GR) (Table 9 and Table 10). In all assays, the highest AA (lowest EC_50_) was found for CS, in both WH and GR. The analysis of variance showed that the part of the leek had the strongest influence on the antioxidant activity expressed by ABTS, DPPH and RED, while in the CHEL assay the method of drying (lower values F-test, Table 10). Bernaert et al. [4] statistically significant correlation between TPC and antioxidant activity of the GR and WH of the leek expressed by free radical scavenging activities against peroxyl (ORAC) and reducing capacity (FRAP). However, they did not find a significant relation between TPC and DPPH assay. Contrary to this study, other authors [3] found a strong and significant correlation between TPC and DPPF both for CR and WH of leek. In our study, we found significant correlations (*p* < 0.05) between TPC and EC_50_ for ABTS, DPPH and RED (r = −0.809, −0.980, and −0.948, respectively).

In most of the cases, drying at higher temperatures caused a decrease in AA in both leek samples, resulting in higher EC_50_ values. Other authors have also observed a similar tendency during AD of leek. The total antioxidant capacity expressed by ferric reducing antioxidant power was decreased about two-fold after dehydration [13]. The GR of leek exhibited stronger AA compared to WH. The highest differences between GR and WH were found in the DPPH assay. A similar tendency was noted by Bernaert et al. [4] when they studied the antioxidant capacity of 30 leek varieties. The highest AA of the green part of leek resulted from the higher content of phenolic compounds as was confirmed by the HPLC analysis.

The ABTS and DPPH assays showed that GR dried at the same temperature (60 °C) using different methods was characterized by different EC_50_ values. The highest values (lowest AA) were found for samples freeze-dried at 60 °C (18.58 and 201.8 mg dm/mL in ABTS and DPPH assays, respectively), while the lowest was found for air-dried leek (17.15 and 184.5 mg dm/mL, respectively). In the case of CHEL and RED assays, the method of drying had only little influence on AA. The same dependence was observed with the drying of WH of leek. The highest EC_50_ values (lowest AA) were found for WH samples freeze-dried at 60 °C (24.29 and 332.4 mg dm/mL in ABTS and DPPH assays, respectively), and the lowest for air-dried samples (22.29 and 308.3 mg dm/mL, respectively). The lowest AA found in WH and GR samples dried at 60 °C might have probably resulted from the longer DT during FD. Additionally, other authors found that both drying temperature and drying time are the crucial factors that influence the antioxidant activity of leek [13] and other vegetables [55,56]. FD is considered to be one of the best but most expensive methods of food dehydration. In industrial practice, the duration and costs of FD are often reduced by increasing the drying temperature or different method of pretreatments, and as a result, the time of dehydration is reduced significantly [57]. However, based on the obtained results, it can be stated that if a higher temperature is used for drying, the lyophilized product will have favorable organoleptic properties such as color and appearance compared to convective-dried sample, but the extent of degradation of bioactive compounds will be similar to that of hot AD. 

## 4. Conclusions

The results of the two-factor analysis of variance showed that both the drying method and the sample (GR or WH) had a significant influence on the determined properties of the dried leek. Additionally, for most of the determined parameters, the interactions between the drying method and the part of the leek were significant. This study proved that the GR of leek contains a higher content of ash and fiber compared to WH. Moreover, it is a valuable source of biologically active compounds, especially phenolic acids. In the dried leek sample, ferulic acid was detected as the dominant phenolic acid. The GR of leek also contained phenolic derivatives, with acylated kaempferol glycoside found to be dominant. The highest level of phenolic acids and flavonoids as well as highest AA and better color preservation was observed in powdered leek samples obtained after freeze-drying at 20 °C (CS). An increase in drying temperature caused the degradation of phenolic compounds and decreased the AA of leek shafts. Most importantly, the application of higher dehydration temperatures for AD, compared to FD and VD, facilitated better preservation of biologically active compounds in the leek sample. This finding is of high importance because in industries, food is often freeze-dried or vacuum-dried at higher temperatures. Although the use of high temperatures during FD or VD allows for better color preservation and shortening the duration of the process, it can also lead to a higher degree of degradation of biologically active compounds. The study demonstrated that GR of freeze-dried leek dried at 20 °C can be a valuable food additive. However, if higher temperatures are to be used, then AD is a better and less cost-effective method, compared to FD and VD.

## Figures and Tables

**Table 1:** The analysis of variance for the drying time of leek.

Parameter	Source of Variance	Sum of Squares	Degrees of Freedom	Mean Square	F–Test	*p*-Value
DT	Intercept	5,544,971	1	5,544,971	61,696.47	0.000001
	SA	1204	1	1204	13.40	0.002112
	DM	4,478,873	3	1,492,958	16,611.49	0.000001
	SA∙DM	294	3	98	1.09	0.381860

DT—drying time, SA—leek sample, DM—drying method.

**Table 2:** Basic chemical composition of fresh and dried leek.

MD *	PL	Dry Matter (%)	Raw Ash (%)	Protein (%)	Raw Fat (%)	Raw Fibre (%)
FL	WH	12.44 ± 0.036 ^a^**	0.95 ± 0.04 ^a^	1.4 ± 0.06 ^a^	0.22 ± 0.008 ^a^	1.42 ± 0.024 ^a^
	GR	11.85 ± 0.088 ^b^	1.12 ± 0.06 ^b^	1.5 ± 0.08 ^a^	0.34 ± 0.011 ^b^	1.85 ± 0.033 ^b^
CS	WH	88.52 ± 0.187 ^b^	4.86 ± 0.125 ^c^	12.1 ± 0.25 ^b^	1.25 ± 0.036 ^c^	5.03 ± 0.101 ^c^
	GR	88.39 ± 0.145 ^b^	5.18 ± 0.137 ^d^	12.3 ± 0.28 ^bc^	2.27 ± 0.042 ^d^	8.16 ± 0.146 ^d^
FD	WH	88.64 ± 0.217 ^b^	4.83 ± 0.121 ^c^	12.2 ± 0.26 ^b^	1.28 ± 0.042 ^c^	5.09 ± 0.121 ^c^
	GR	88.43 ± 0.112 ^b^	5.12 ± 0.142 ^d^	12.4 ± 0.31 ^bc^	2.25 ± 0.048 ^d^	8.20 ± 0.173 ^d^
VD	WH	88.82 ± 0.243 ^b^	4.77 ± 0.157 ^c^	11.8 ± 0.17 ^b^	1.29 ± 0.029 ^c^	4.99 ± 0.145 ^c^
	GR	88.74 ± 0.211 ^b^	5.24 ± 0.180 ^d^	12.1 ± 0.19 ^b^	2.23 ± 0.035 ^d^	8.37 ± 0.229 ^d^
AD	WH	88.78 ± 0.308 ^b^	4.74 ± 0.112 ^c^	12.2 ± 0.15 ^b^	1.24 ± 0.042 ^c^	5.03 ± 0.192 ^c^
	GR	88.63 ± 0.282 ^b^	5.09 ± 0.146 ^d^	12.7 ± 0.20 ^c^	2.28 ± 0.056 ^d^	8.41 ± 0.278 ^d^

* MD—method of drying, PL—part of leek, FL—fresh leek, CS—control sample, FD—freeze-drying, VD—vacuum drying, AD—air drying, WH—white shaft of leek, GR—green shaft of leek; ** The values designated by the different small letters in the columns of the table are significantly different (α = 0.05).

**Table 3:** Effect of the drying method on the color parameters of leek.

MD *	PL	L*	a*	b*
CS	WH	85.79 ± 0.85 ^a^**	−3.44 ± 0.17 ^a^	22.15 ± 0.48 ^b^
	GR	76.22 ± 0.54 ^b^	−9.32 ± 0.10 ^f^	27.07 ± 0.29 ^c^
FD	WH	85.04 ± 1.48 ^a^	−3.09 ± 0.20 ^ab^	21.44 ± 0.62 ^b^
	GR	73.58 ± 0.80 ^c^	−8.96 ± 0.09 ^g^	29.05 ± 0.29 ^d^
VD	WH	83.46 ± 1.99 ^a^	−2.65 ± 0.26 ^b^	16.15 ± 1.26 ^a^
	GR	74.17 ± 0.57 ^c^	−7.65 ± 0.08 ^e^	28.46 ± 0.27 ^d^
AD	WH	84.62 ± 0.40 ^a^	−1.24 ± 0.05 ^c^	17.35 ± 0.61 ^a^
	GR	70.98 ± 1.54 ^c^	−7.14 ± 0.09 ^d^	26.38 ± 0.83 ^c^

* MD—method of drying, PL—part of leek, CS—control sample, FD—freeze-drying, VD—vacuum drying, AD—air drying, WH—white shaft of leek, GR—green shaft of leek, L*—lightness, a*—greenness, b*—yellowness, ** The values designated by the different small letters in the columns of the table are significantly different (α = 0.05).

**Table 4:** The analysis of variance for parameters characterizing the color of dried leek.

Parameter	Source of Variance	Sum of Squares	Degrees of Freedom	Mean Square	F—Test	*p*-Value
L*	Intercept	150,671.7	1	150,671.7	118,077.4	0.000001
	SA	660.1	1	660.1	517.3	0.000001
	DM	32.2	3	10.7	8.4	0.001388
	SA∙DM	32.9	3	11.0	8.6	0.001247
a*	Intercept	709.7025	1	709.7025	31,724.46	0.000001
	SA	192.4967	1	192.4967	8604.81	0.000001
	DM	17.2485	3	5.7495	257.01	0.000001
	SA∙DM	0.8738	3	0.2913	13.02	0.000146
b*	Intercept	13,261.05	1	13,261.05	32,360.47	0.000001
	SA	430.36	1	430.36	1050.19	0.000001
	DM	50.18	3	16.73	40.82	0.000001
	SA∙DM	42.46	3	14.15	34.54	0.000001

DM—drying method, SA—leek sample.

**Table 5:** The analysis of variance for the total phenolic content in dried leek.

Parameter	Source of Variance	Sum of Squares	Degrees of Freedom	Mean Square	F—Test	*p*-Value
DT	Intercept	10,834.10	1	10,834.10	5626.752	0.000001
	SA	9266.15	1	9266.15	4812.430	0.000001
	DM	213.79	3	71.26	37.011	0.000001
	SA∙DM	270.28	3	90.09	46.790	0.000001

DT—drying time, SA—leek sample, DM—drying method.

**Table 6:** Identified and marked phenolic acids in a white and green shaft of the leek.

MD *	PL	Average (µg/mg dm)					
		PRO	POH	CAF	PCO	FER	SIN
CS	WH	0.09 ± 0.00 ^bc^**	<BQL	<BQL	0.91 ± 0.03 ^b^	<BQL	0.29 ± 0.02 ^b^
	GR	0.20 ± 0.01 ^f^	0.15 ± 0.01 ^b^	0.31 ± 0.03 ^b^	5.15 ± 0.18 ^f^	42.92 ± 1.84 ^e^	1.82 ± 0.07 ^f^
FD	WH	0.10 ± 0.01 ^c^	0.05 ± 0.00 ^a^	<BQL	0.66 ± 0.03 ^a^	<BQL	0.26 ± 0.03 ^ab^
	GR	0.16 ± 0.02 ^d^	<BQL	0.05 ± 0.01 ^a^	3.94 ± 0.11 ^d^	32.14 ± 0.94 ^c^	0.55 ± 0.02 ^d^
VD	WH	0.08 ± 0.00 ^b^	0.24 ± 0.01 ^d^	<BQL	0.80 ± 0.02 ^ab^	<BQL	0.18 ± 0.01 ^a^
	GR	0.17 ± 0.02 ^de^	0.20 ± 0.03 ^c^	0.72 ± 0.03 ^d^	3.88 ± 0.29 ^d^	35.79 ± 3.32 ^d^	1.54 ± 0.11 ^e^
AD	WH	0.06 ± 0.00 ^a^	0.15 ± 0.01 ^b^	<BQL	2.00 ± 0.12 ^c^	0.16 ± 0.03 ^a^	0.38 ± 0.02 ^c^
	GR	0.18 ± 0.01 ^e^	0.15 ± 0.01 ^b^	0.50 ± 0.05 ^c^	4.52 ± 0.22 ^e^	26.71 ± 1.66 ^b^	1.88 ± 0.10 ^f^

* MD—method of drying, PL—part of leek, CS—control sample, FD—freeze-drying, VD—vacuum drying, AD—air drying, WH—white shaft of leek, GR—green shaft of leek, Phenolic acids: PRO—protocatechuic, POH—*p*-hydroxybenzoic, CAF—caffeic, PCO—*p*-coumaric, FER—ferulic, SIN—synapic, BQL—below the limit of quantification; ** The values designated by the different small letters in the columns of the table are significantly different (α = 0.05).

**Table 7:** The analysis of variance for parameters characterizing the phenolic acids of dried leek.

Parameter	Source of Variance	Sum of Squares	Degrees of Freedom	Mean Square	F—Test	*p*-Value
PRO	Intercept	0.397056	1	0.397056	2329.779	0.000001
	SA	0.055651	1	0.055651	326.541	0.000001
	DM	0.002625	3	0.000875	5.135	0.011201
	SA∙DM	0.004675	3	0.001558	9.143	0.000929
POH	Intercept	0.330717	1	0.330717	2239.002	0.000001
	SA	0.001183	1	0.001183	8.008	0.012076
	DM	0.130844	3	0.043615	295.277	0.000001
	SA∙DM	0.038722	3	0.012907	87.385	0.000001
CAF	Intercept	0.931331	1	0.931331	1566.923	0.000001
	SA	0.931331	1	0.931331	1566.923	0.000001
	DM	0.364684	3	0.121561	204.522	0.000001
	SA∙DM	0.364684	3	0.121561	204.522	0.000001
PCO	Intercept	7112.547	1	7112.547	3152.571	0.000001
	SA	7080.152	1	7080.152	3138.212	0.000001
	DM	204.399	3	68.133	30.199	0.000001
	SA∙DM	211.630	3	70.543	31.268	0.000001
FER	Intercept	7112.547	1	7112.547	3152.571	0.000001
	SA	7080.152	1	7080.152	3138.212	0.000000
	DM	204.399	3	68.133	30.199	0.000001
	SA∙DM	211.630	3	70.543	31.268	0.000001
SIN	Intercept	17.86592	1	17.86592	5155.794	0.000001
	SA	8.18916	1	8.18916	2363.249	0.000001
	DM	1.89709	3	0.63236	182.489	0.000001
	SA∙DM	1.57736	3	0.52579	151.733	0.000001

Phenolic acids: PRO—protocatechuic, POH—*p*-hydroxybenzoic, CAF—caffeic, PCO—*p*-coumaric, FER—ferulic, SIN—synapic, BQL—below the limit of quantification; DM—drying method, SA—leek sample.

**Table 8:** Flavonoid contents I–X (kaempferol derivatives) in white and green shafts of leek.

Average (mg/g dm)	PL	MD *			
		CS	FD	VD	AD
I	WH	(-)	(-)	(-)	(-)
	GR	0.052 ± 0.004 ^a^**	0.070 ± 0.005 ^c^	0.055 ± 0.004 ^a^	0.062 ± 0.002 ^b^
II	WH	(-)	(-)	(-)	(-)
	GR	0.748 ± 0.033 ^d^	0.295 ± 0.020 ^b^	0.158 ± 0.005 ^a^	0.403 ± 0.007 ^c^
III	WH	(-)	(-)	(-)	(-)
	GR	0.089 ± 0.004 ^b^	0.036 ± 0.002 ^a^	<BQL	0.093 ± 0.004 ^b^
IV	WH	(-)	(-)	(-)	(-)
	GR	<BQL	0.057 ± 0.003 ^a^	0.051 ± 0.008 ^a^	0.055 ± 0.002 ^a^
V	WH	(-)	(-)	(-)	(-)
	GR	0.173 ± 0.005 ^b^	0.235 ± 0.022 ^c^	< BQL	0.127 ± 0.003 ^a^
VI	WH	(-)	(-)	(-)	(-)
	GR	1.038 ± 0.030 ^c^	0.501 ± 0.034 ^b^	0.221 ± 0.005 ^a^	0.525 ± 0.011 ^b^
VII	WH	(-)	(-)	(-)	(-)
	GR	0.312 ± 0.009 ^c^	0.114 ± 0.006 ^a^	0.467 ± 0.007 ^d^	0.182 ± 0.008 ^b^
VIII	WH	(-)	(-)	(-)	(-)
	GR	0.181 ± 0.009 ^c^	0.039 ± 0.001 ^a^	<BQL	0.171 ± 0.009 ^b^
IX	WH	(-)	(-)	(-)	(-)
	GR	0.066 ± 0.001 ^c^	0.031 ± 0.002 ^a^	<BQL	0.057 ± 0.002 ^b^
X	WH	(-)	(-)	(-)	(-)
	GR	0.303 ± 0.013 ^c^	0.107 ± 0.008 ^a^	0.223 ± 0.007 ^b^	0.235 ± 0.012 ^b^

* MD—method of drying, PL—part of leek, CS—control sample, FD—freeze-drying, VD—vacuum drying, AD—air drying, WH—white shaft of leek, GR—green shaft of leek, The flavonoids compounds were identification: I, IV—kaempferol dihexoside, II, VIII, IX—kaempferol derivative, III—kaempferol glycoside, V, VI, VII, IX—acylated kaempferol glycoside, (-)—not detected, <BQL—below the limit of quantification, ** The values designated by the different small letters in the columns of the table are significantly different (α = 0.05).

**Table 9:** Antioxidant activity of dried white and green shaft of leek.

MD *	PL	Antioxidant Activity			
		ABTS (EC_50_; mg dm/mL)	DPPH (EC_50_; mg dm/mL)	CHEL (EC_50_; mg dm/mL)	RED (EC_50_; mg dm/mL)
CS	WH	18.69 ± 0.48 ^b^**	289.6 ± 7.37 ^e^	35.36 ± 0.53 ^ab^	16.72 ± 0.32 ^d^
	GR	16.51 ± 0.87 ^a^	126.8 ± 3.61 ^a^	35.13 ± 0.87 ^a^	6.98 ± 0.80 ^a^
FD	WH	24.29 ± 0.26 ^e^	332.4 ± 8.91 ^f^	38.50 ± 1.16 ^e^	17.99 ± 0.23 ^e^
	GR	18.58 ± 1.02 ^b^	201.8 ± 4.25 ^c^	36.06 ± 0.65 ^bc^	13.26 ± 0.42 ^c^
VD	WH	21.99 ± 0.46 ^d^	313.4 ± 7.66 ^e^	38.54 ± 1.29 ^c^	18.89 ± 0.37 ^f^
	GR	18.39 ± 0.71 ^b^	185.0 ± 5.13 ^b^	36.50 ± 0.67 ^ac^	10.65 ± 0.47 ^b^
AD	WH	22.29 ± 0.59 ^d^	308.3 ± 9.49 ^e^	39.81 ± 0.97 ^e^	18.30 ± 0.68 ^ef^
	GR	17.15 ± 0.50 ^a^	184.5 ± 6.23 ^b^	37.90 ± 1.08 ^d^	13.65 ± 0.44 ^dc^

* MD—method of drying, PL—shaft of leek, FL—fresh leek, FD—freeze-drying, VD—vacuum drying, AD—air drying, WH—white shaft of leek, GR—green shaft of leek, ABTS—antioxidant activity, DPPH—antioxidant activity, CHEL—chelating ability, RED—ability to reduce. ** The values designated by the different small letters in the columns of the table are significantly different (α = 0.05).

**Table 10:** The analysis of variance for parameters characterizing antioxidant properties of dried leek.

Parameter	Source of Variance	Sum of Squares	Degrees of Freedom	Mean Square	F—Test	*p*-Value
ABTS	Intercept	9378.888	1	9378.888	23,687.30	0.000001
	SA	102.259	1	102.259	258.26	0.000001
	DM	47.070	3	15.690	39.63	0.000001
	SA∙DM	11.450	3	3.817	9.64	0.000715
DPPH	Intercept	1,416,170	1	1,416,170	33,947.01	0.000001
	SA	111,250	1	111,250	2666.76	0.000001
	DM	10,862	3	3621	86.79	0.000001
	SA∙DM	1366	3	455	10.91	0.000379
CHEL	Intercept	33,553.79	1	33,553.79	37,367.45	0.000001
	SA	10.45	1	10.45	11.64	0.003568
	DM	40.34	3	13.45	14.97	0.000066
	SA∙DM	4.66	3	1.55	1.73	0.200831
RED	Intercept	5071.843	1	5071.843	22,831.14	0.000001
	SA	285.591	1	285.591	1285.60	0.000001
	DM	66.321	3	22.107	99.52	0.000001
	SA∙DM	30.000	3	10.000	45.02	0.000001

DT—drying time, SA—leek sample, DM—drying method, ABTS—antioxidant activity, DPPH—antioxidant activity, CHEL—chelating ability, RED—ability to reduce.

## Data Availability

The data presented in this study are available on request from the corresponding author.
